# Novolac-based poly(1,2,3-triazolium)s with good ionic conductivity and enhanced CO_2_ permeation†

**DOI:** 10.1039/c8ra00541a

**Published:** 2018-02-26

**Authors:** Lvyuan Ye, Liqiang Wan, Junkun Tang, Yujing Li, Farong Huang

**Affiliations:** Key Laboratory of Specially Functional Polymeric Materials and Related Technology, School of Materials Science and Engineering, East China University of Science and Technology, Ministry of Education Shanghai 200237 China fhuanglab@ecust.edu.cn +86-021-64251110

## Abstract

Novolac-based poly(1,2,3-triazolium)s with 1,2,3-triazolium side groups spaced by oligo(ethylene glycol), a new kind of poly(ionic liquid) membrane, was prepared *via* the well-known Click chemistry (1,3-dipolar cycloaddition reaction). The thermal properties, ionic conductivity and gas permeation performance of these self-standing membranes were investigated. The obtained membranes exhibit glass transition temperatures ranging from −1 °C to −7.5 °C, and a temperature at 10% weight loss above 330 °C. These membranes have good ionic conductivity (*σ*_DC_ up to 5.1 × 10^−7^ S cm^−1^ at 30 °C under anhydrous conditions) as compared with the reported 1,2,3-triazolium-based crosslinked polymers. And they could be potentially used for CO_2_ separation as they exhibit enhanced CO_2_ permeability up to 434.5 barrer at 4 atm pressure.

## Abstract 2

Novolac-based poly(1,2,3-triazoliums)s with 1,2,3-triazolium in the side groups spaced by oligo(ethylene glycol) show enhanced CO_2_ permeability.

## Graphical abstract

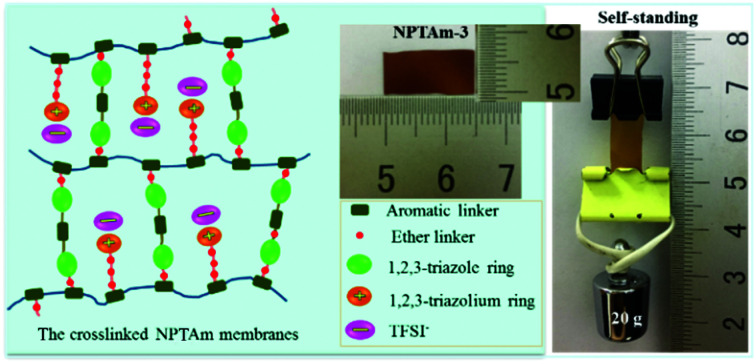

## Introduction

Poly(ionic liquid)s (PILs) ideally contain many combined properties of ILs (*e.g.* tunable solubility, thermal stability, ionic conductivity, *etc.*) with intrinsic polymer properties (*e.g.* processability, adhesion, film-forming properties, *etc.*).^1^ Since ILs are known to possess high CO_2_ sorption, PIL membranes for CO_2_ permeation applications have gained substantial attention.^2^ PILs are supposed to form tight and stable matrices, which are more energy efficient and environmentally benign compared with the commercial separation materials.^3^ Generally, the IL moieties provide good CO_2_ selectivity and other functional groups (*e.g.* ether segments, aromatic group, *etc.*) in PIL membranes could further improve CO_2_ permeability. PILs membranes with imidazolium or pyrrolidinium cations based on various backbones (*e.g.* polyvinyl, polybenzimidazole, polyurethanes, *etc.*) have already proven their capability in CO_2_ separation.^4–8^ But, the 1,2,3-triazolium cations based PILs (poly(1,2,3-triazolium)s, TPILs) used for gas separation has just emerged.^5,9^

1,2,3-Triazoliums, the ionization products of 1,2,3-triazoles, are attracting great interest as a type of new potential electrolytes for the reason that the well-known “Click” chemistry (Huisgen 1,3-dipolar cycloaddition reaction) renders the syntheses and functionalization of 1,2,3-triazoles easy and flexible. Up to now, numerous TPILs have been reported.^12^ For example, through bringing in triethylene glycol (TEG) spacers, main-chain TPILs with bis(trifluoromethylsulfonyl)imide (TFSI^−^) anions have shown relatively high ionic conductivity (2.0 × 10^−5^ S cm^−1^) with a low glass transition temperature (*T*_g_) of −35 °C.^10^ Hyperbranched TPILs with oligo(ethylene glycol) (OEG) terminal groups exhibited ionic conductivity 7.7 × 10^−6^ S cm^−1^ with *T*_g_ of −14.9 °C.^11^ Several side-chain TPILs were also investigated, for instance, poly(vinyl ester 1,2,3-triazolium) with TFSI^−^ anions displayed ionic conductivity of 9.2 × 10^−7^ S cm^−1^ with *T*_g_ of −16 °C while polyacrylates with 1,2,3-triazolium side groups spaced by TEG groups displayed higher ionic conductivity of 1.1 × 10^−5^ S cm^−1^ with lower *T*_g_ of −36 °C.^12,13^ It can be concluded that the TFSI^−^ is the best candidate anion for conductive materials, meanwhile, the introduction of 1,2,3-triazolium in the side groups spaced by rich ether groups are effective ways to improve the ionic conductivity of the obtained TPILs. Unfortunately, some of the previously reported TPILs were unable to be used as gas separation membranes due to their brittle or viscous nature resulting from the flexible polymer structure.

To promote innovative applications in diverse fields, 1,2,3-triazolium-based crosslinked polymers are emerging.^14,15^ For example, 1,2,3-triazolium-based epoxy-amine networks and polyether-based 1,2,3-triazoliums were recently reported and both of them show satisfying ionic conductivity (up to 2.0 × 10^−7^ S cm^−1^, 10^−6^ S cm^−1^, separately).^9,16^ However, only the latter have been evaluated as gas separation membranes, for the first time. In view of the fact that innovation and breakthrough is still in need in both the crosslinked TPILs and their applications in CO_2_ separation fields, we attempt to design and prepare new self-standing crosslinked TPILs membranes by introducing rigid benzene rings and 1,2,3-triazoles in the polymer structure.

In this contribution, we report novolac-based poly(1,2,3-triazolium)s (NPTAm) membranes with novolac-based poly(1,2,3-triazole)s as backbone, together with 1,2,3-triazolium in side groups spaced by OEG, containing TFSI^−^ as counter-anion. Thermal, ion-conducting properties and CO_2_ permeability of these membranes were investigated.

## Experimental

### Materials

PN resin, *p*-xylyene diazide and azido-2-(2-(2-methoxyethoxy)ethoxy)ethane (OEG-N_3_) were prepared according to the literature, separately.^17–19^ Sodium ascorbate (99%) and CuSO_4_·5H_2_O (99%) were purchased from Shanghai Chemical Reagents Company. Iodomethane (CH_3_I, 99%) was purchased from Shanghai Dibo Chemical Reagents Company. Ethylenediaminetetraacetic acid disodium salt (EDTA), lithium bis(trifluoromethanesulfonyl)imide (LiTFSI), and other reagents and solvents were purchased from Adamas and used as received.

### Synthesis of PN-OEG through CuAAC

To a solution of PN resin (3.17 g, 20 mmol of alkyne group) and OEG-N_3_ (1.39 g, 8 mmol) in DMF (50 mL), CuSO_4_·5H_2_O aqueous solution (0.8 mmol in 1.2 mL of H_2_O) was added. After bubbling nitrogen for 30 min, fresh sodium ascorbate solution (2.4 mmol in 0.8 mL H_2_O) was added. The resulting mixture was stirred for 48 h at 55 °C. After evaporation of the solvent under reduced pressure, the mixture was poured into H_2_O, then extracted with DCM (50 mL), and the organic layer was washed with EDTA aqueous solution and saturated NaCl aqueous solution until the aqueous phase became colourless. After dried by MgSO_4_, the organic layer was concentrated and precipitated three times with diethyl ether, then dried in vacuum and a reddish brown solid PN-OEG (3.88 g, yield 85%) was obtained. ^1^H NMR (400 MHz, DMSO, ppm): *δ* 8.11–7.94 (m, triazole-*H*), 7.08–6.70 (m, aromatic-*H*), 5.02 (s, –N–N=NCH_2_C*H*_2_O–), 4.71 (s, –O–C*H*_2_–C≡C–), 4.48 (s, –C–C*H*_2_–C, –N=N–NC*H*_2_CH_2_–O–, –N=N–NCH_2_C*H*_2_–O–), 3.71 (m, –C*H*_2_O–), 3.37 (s, –C≡C*H*), 1.00 (s, –CH_2_C*H*_3_).

### Synthesis of [PN-OEG]^+^I^−^

To a solution of PN-OEG (1.71 g, 3 mmol of triazole groups) in 50 mL CH_3_CN, CH_3_I (1.42 g, 10 mmol) was added, and the mixture was stirred at 45 °C for 3 d. The mixture was concentrated and precipitated three times in diethyl ether and dried in vacuum to get [PN-OEG]^+^I^−^ (2.03 g, yield 95%) as a yellow solid. ^1^H NMR (400 MHz, DMSO, ppm): *δ* 9.01 (s, triazolium-*H*), 7.08–6.70 (m, aromatic-*H*), 5.44 (s, –N–N=NCH_2_C*H*_2_O–), 4.86–4.71 (m, –O–C*H*_2_–C≡C–), 4.35 (m, –NC*H*_3_), 4.16 (s, –CC*H*_2_C–), 3.93 (m, –N=N–NC*H*_2_CH_2_O–, –N=N–NCH_2_C*H*_2_–O–), 3.55 (m, –C*H*_2_O–), 3.37 (s, –C≡C*H*), 1.00 (s, –CH_2_C*H*_3_).

### Synthesis of [PN-OEG]^+^TFSI^−^

A solution of [PN-OEG]^+^I^−^ (1.78 g, 2.5 mmol of 1,2,3-triazole groups) and LiTFSI (2.15 g, 7.5 mmol) in a mixture of acetone (35 mL) and methanol (35 mL) was stirred at 45 °C for 2 d. The heterogeneous mixture was concentrated and precipitated in deionized water several times, until no AgI precipitate generated when the deionized water was tested with AgNO_3_, then dried in vacuum, and a reddish brown viscous material [PN-OEG]^+^TFSI^−^ (1.30 g, yield 60%) was obtained. ^1^H NMR (400 MHz, DMSO, ppm): *δ* 8.95 (s, triazolium-*H*), 7.00–6.84 (m, aromatic-*H*), 5.36 (s, –N–N=NCH_2_C*H*_2_O–), 4.83 (m, –O–C*H*_2_–C≡C–), 4.31 (s, –NC*H*_3_), 3.91 (m, –CC*H*_2_C–, –N=N–NC*H*_2_CH_2_–O–, –N=N–NCH_2_C*H*_2_–O–) 3.56 (m, –C*H*_2_O–), 3.37 (s, –C≡C*H*), 1.00 (s, –CH_2_C*H*_3_). ^19^F NMR (400 MHz, DMSO, ppm): *δ* −78.8.

### Preparation of the crosslinked NPTAm membrane

A stoichiometric mixture of [PN-OEG]^+^TFSI^−^ (1.15 g, 2 mmol of alkyne) and *p*-xylyene diazide (0.19 g, 1 mmol) was dissolved in DMF (5 g), and then was stirred at 70 °C for 2 h, following by casting the concentrated mixture onto a glass plate and levelling it with a stainless steel scraper, which had been preheated to 70 °C. The glass plate was placed onto a horizontal platform. Then, the system was sequentially cured (70 °C/3 h + 80 °C/3 h + 120 °C/2 h + 150 °C/4 h). After that, the heating oven was turned off and the whole system was gradually cooled to room temperature. The membrane, named as NPTAm-1, was obtained by immersing the glass plate in water and was then dried at 100 °C for 0.5 h for further use.

By changing the feed molar ratio of PN with OEG-N_3_ (1 : 0.6 and 1 : 0.8) when initially synthesizing PN-OEG, NPTAm-2 and NPTAm-3 membranes were separately obtained (ESI†).

### Characterization

#### Spectroscopic and thermal characterizations

All NMR spectra data were obtained on a Bruker Advance 400 MHz Spectrometer (Bruker, USA) using tetramethylsilane (TMS) as an internal standard in DMSO-*d*_6_. FT-IR spectrum measurements were carried out on a Nicolet iS10 FTIR spectrophotometer (Thermo Scientific, USA) in the region of 4000–400 cm^−1^ using KBr pellets. TGA were conducted on a TGA/DSC 1 (Mettler Toledo, Switzerland) under nitrogen at a heating rate of 10 °C min^−1^. DSC was performed in a nitrogen atmosphere on a TA Q2000 analyser (TA, USA). The samples were first heated from 40 °C to 150 °C and held at 150 °C for 2 min to eliminate the thermal history, then cooled to −30 °C, and finally heated again from −30 °C to 150 °C. The heating or cooling rate remained 20 °C min^−1^ and *T*_g_ values were recorded during the second heating cycle.

#### Ionic conductivity measurements

The ionic conductivity was measured using a high-resolution Alpha-Analyzer (BDS, Novocontrol GmbH, Germany) assisted by a Quatro temperature controller under nitrogen. The samples were placed between two polished brass electrodes and heated at 110 °C for 4 h under a flow of pure nitrogen. At the same time, the dielectric properties were measured to monitor the equilibration process of the sample. Frequency sweeps were then performed isothermally from 10 MHz to 0.1 Hz by applying a sinusoidal voltage of 0.1 V ranging from 110 °C to −30 °C in steps of 20 °C. The temperature was controlled by heating the sample under a flow of pure nitrogen, which could exclude oxygen and humidity in the test chamber.

#### Gas permeation measurements

The gas permeation properties of the membranes were measured by a standard variable volume method at upstream gas pressure of 4 atm pressure at 25 °C according to the literature^20^ (Fig. S1†).

The gas permeability (*P*) was determined from eqn (1):1
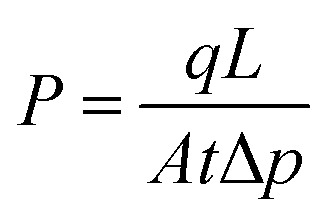
where *q* is the infiltration capacity of the gas passing through the membrane, *L* is the membrane thickness, Δ*p* is the differential pressure of feed and permeate side and *A* is the effective membrane area.

The CO_2_/N_2_ selectivity (*α*_CO_2_/N_2__) was calculated from eqn (2):2
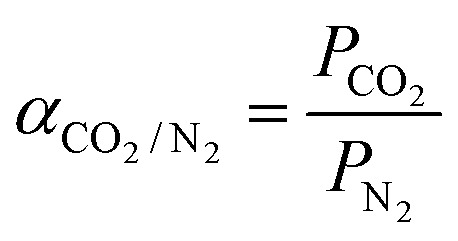
where *P*_CO_2__ is the gas permeability of CO_2_ and *P*_N_2__ is the gas permeability of N_2_. The data averaged from 3 samples for each membrane.

## Results and discussion

### Preparation of NPTAm membranes through CuAAC reaction and 1,3-dipolar cycloaddition

**Fig. 1:**
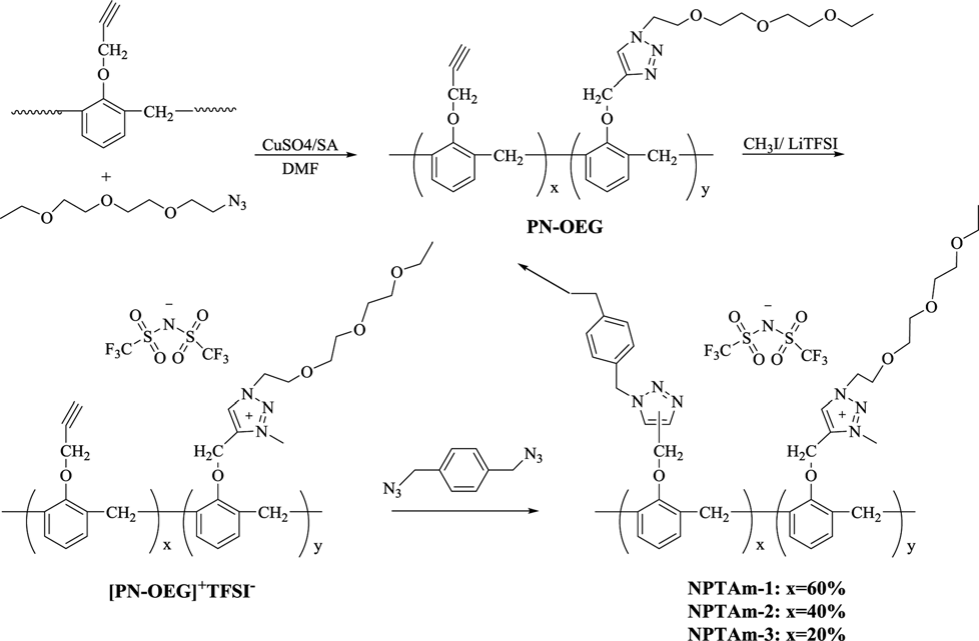
Syntheses of the crosslinked NPTAm membranes and the self-standing ability test.

As shown in Fig. 1, PN-OEG was firstly obtained through Cu(i)-catalyzed azide–alkyne cycloaddition reaction (Click chemistry) by adding the catalyst of CuSO_4_/NaVc to the solution of PN and OEG-N_3_ in DMF. Next, PN-OEG was alkylated by CH_3_I to get the triazolium iodide polymers, [PN-OEG]^+^I^−^, following an anion metathesis reaction performed by exchanging the iodide anion (I^−^) with TFSI^−^. After being washed through precipitating the mixture into deionized water for several times, no AgI formed when the water phase was tested with AgNO_3_, which showed that the generated LiI had been fully removed, and [PN-OEG]^+^TFSI^−^ was obtained. Membrane fabrication is based on Huisgen 1,3-dipolar cycloaddition between *p*-xylyene diazide and [PN-OEG]^+^TFSI^−^ and the membrane was cured to 150 °C to ensure the complete polymerization. Three different membranes were prepared by manipulate the ratio between PN and OEG-N_3_ as 1 : 0.4, 1 : 0.6 and 1 : 0.8, and thus the number of side-chain 1,2,3-triazolium and OEG moieties together with the crosslinking density were easily changed. The obtained membranes were observed to be self-standing as shown in Fig. 1. NPTAm-3 membrane (thickness 150 μm, wide 0.7 cm), as an example, could easily sustained an applied load of at least 20 g, that is, tensile force of a minimum of 1.87 × 10^5^ Pa.

### Structural characterization

**Fig. 2:**
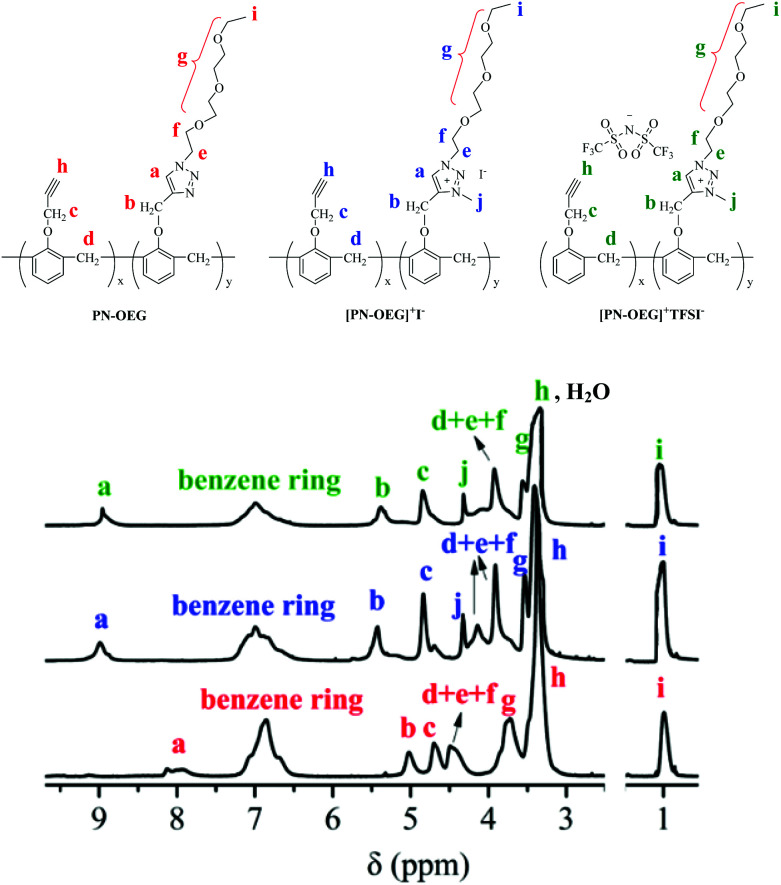
^1^H NMR spectra (25 °C, DMSO-*d*_6_) of PN-OEG, [PN-OEG]^+^I^−^ and [PN-OEG]^+^TFSI^−^.

Fig. 2 shows the ^1^H NMR spectra of PN-OEG, [PN-OEG]^+^I^−^ and [PN-OEG]^+^TFSI^−^. For PN-OEG, the signals at 8.11–7.94 ppm assigned to 1,2,3-triazole ring confirm the success of CuAAC reaction. After alkylated by CH_3_I, the triazole signals moves down to 9.01 ppm in the spectra of [PN-OEG]^+^I^−^, clearly demonstrating the accomplishment of quaternization and the formation of 1,2,3-triazolium. Also, the new signals appeared at 4.35 ppm could be ascribed to the methyl protons connecting with the nitrogen atom of the 1,2,3-triazolium ring. After exchanging I^−^ anions with TFSI^−^ anions, in the spectra of [PN-OEG]^+^TFSI^−^, the chemical shift of 1,2,3-triazolium protons slightly shifted from 9.01 ppm to 8.95 ppm and the signal of methyl protons shifted down to 4.31 ppm, indicating quantitative anion-exchange reaction. Additionally, ^19^F NMR spectra of [PN-OEG]^+^TFSI^−^ (Fig. S2†) clearly showed a single peak, which was further evidence for the completion of anion exchange reaction.

FTIR analysis was used to investigate the reaction between *p*-xylyene diazide and [PN-OEG]^+^TFSI^−^ (Fig. S3†). Compared to the spectrum of [PN-OEG]^+^TFSI^−^, the red shift of the 1,2,3-triazole absorption peak from 3120 cm^−1^ to 3128 cm^−1^ and the disappearance of the –C≡C– group absorption peak at 2122 cm^−1^ are indicative of the formation of NPTAm-1 through the polymerization based on 1,3-dipolar cycloaddition reaction between the alkyne groups in [PN-OEG]^+^TFSI^−^ and the azido groups in *p*-xylyene diazide.

### Thermal properties of the NPTAm membranes

**Fig. 3:**
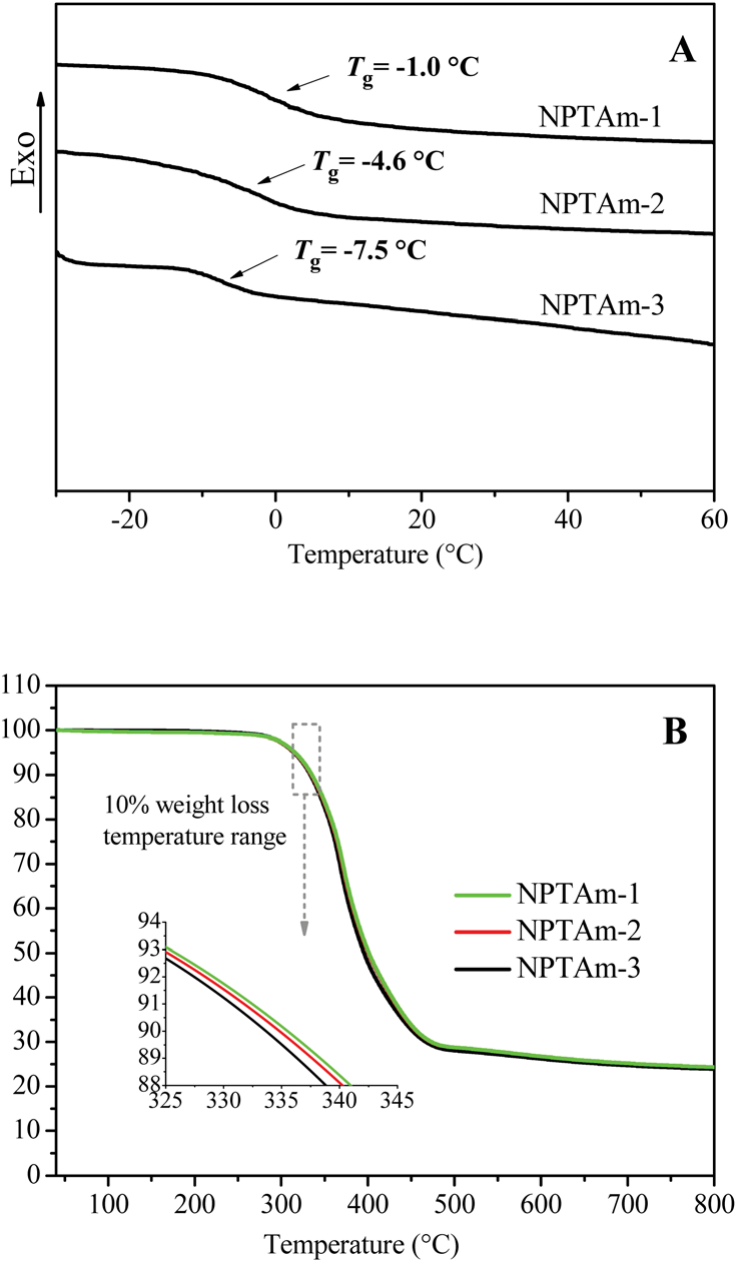
(A) DSC, (B) TGA curves of the crosslinked NPTAm membranes.

Generally, ion transport in polyelectrolytes is related with segmental motion in the vicinity of conducting ions, and a low *T*_g_ promotes the transportation of ions.^11,21^ The thermal properties of the NPTAm membranes were investigated by DSC and TGA (Fig. 3, Table 1). All the samples exhibit a single transition corresponding to the glass transition temperature (*T*_g_) values of −1.0 °C for NPTAm-1, −4.6 °C for NPTAm-2 and −7.5 °C for NPTAm-3. NPTAm-3 showed the lowest *T*_g_, indicating the lowest crosslinking density and inversely most flexible oligo(ethylene glycol) side groups. It could be further confirmed from the DMA result (Fig. S4†) that NPTAm-3 exhibited the lowest storage modulus in the rubbery state (*E*′ = 16.9 MPa and 5.2 MPa at 25 °C for NPTAm-1 and NPTAm-3, respectively). However, these NPTAm membranes showed remarkably high *T*_g_ values as compared with previously reported crosslinked TPILs having TFSI^−^ counter-anions (*T*_g_ ranges from −52 to −65 °C).^9,16^ The rigid benzene rings and 1,2,3-triazoles in the backbone along with the π–π stacking and hydrogen bonding character of numerous 1,2,3-triazolium groups^22^ may account for the higher *T*_g_ values. TGA results indicate that all the membranes have good thermal stabilities above 330 °C (*T*_d10_), which are in the upper range of values previously reported TPILs (145–371 °C).^5^

**Table 1:** Thermal and ion-conducting properties of the NPTAm membranes

Entry	*T*_g_ a (°C)	*T*_d10_ b (°C)	*E*′^c^ (MPa)	*σ*_DC_ at^d^ 30 °C (S cm^−1^)	*B* d (K)	*T*_0_ d (K)	*σ*_∞_ d (S cm^−1^)
NPTAm-1	−1.0	336	16.9	2.8 × 10^−8^	1400	212	0.113
NPTAm-2	−4.6	335	—	7.9 × 10^−8^	1389	210	0.253
NPTAm-3	−7.5	333	5.2	5.1 × 10^−7^	1349	213	1.832

a Obtained from DSC.

b Obtained from TGA.

c Obtained from DMA.

d Obtained from BDS.

### Ionic conductivity of the NPTAm membranes

**Fig. 4:**
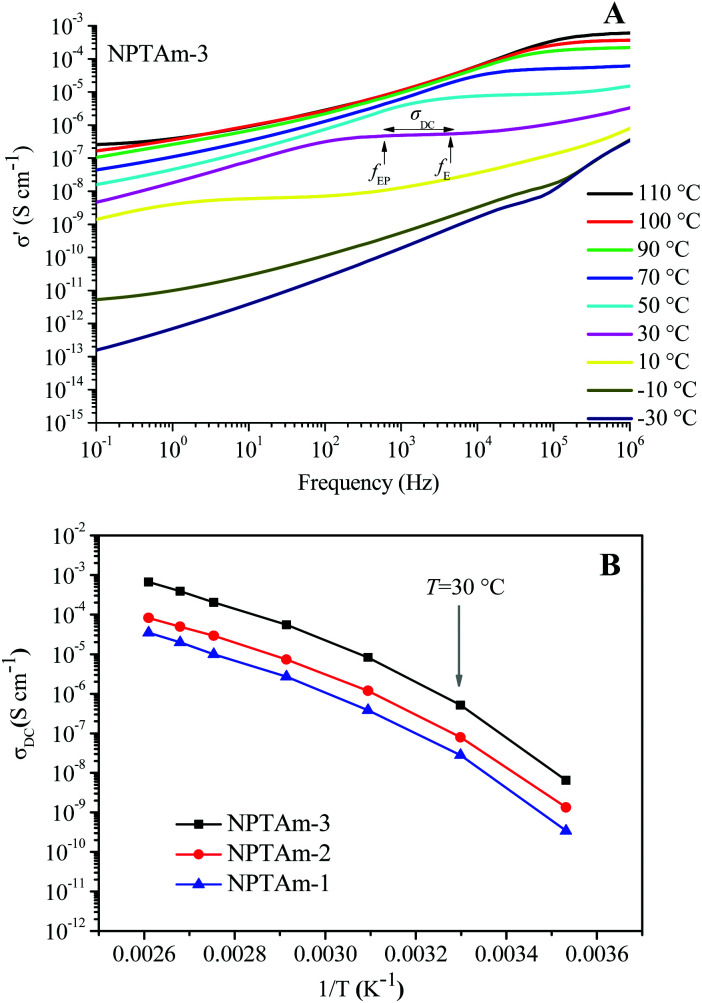
(A) Conductivity *versus* frequency for the crosslinked NPTAm membranes. (B) Direct current conductivity (*σ*_DC_) *versus* inverse temperature for the NPTAm membranes. The solid lines represent the best VFT fit of experimental data using eqn (3).

The temperature dependence of the anhydrous ionic conductivity of these NPTAm membranes was investigated by BDS. As an example, Fig. 4A describes the frequency (*ω*) dependence of the conductivity (*σ*′) at temperature ranging from −30 to 110 °C for NPTAm-3. A plateau in the *σ*′ value between two characteristic frequency (*f*_E_, *f*_EP_) could be observed for all membranes samples when the temperature was above 10 °C (Fig. S5†). This plateau corresponds to the direct current conductivity (*σ*_DC_), which associates with the appearance of the ionic conduction character. Considering the correlation between the charge transport of the ionic species and the molecular mobility of the polymer chain, the evolution of *σ*_DC_ with reciprocal temperature for all NPTAm membranes follows a typical Vogel–Fulcher–Tammann (VFT) dependence, and thus the experimental results fitted with the VFT eqn (3).3
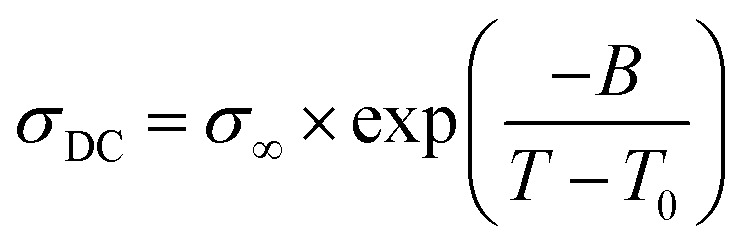
where *σ*_∞_ is the ionic conductivity in the limit of high temperature, *B* is the fitting parameter related to the activation energy of ionic conduction, and *T*_0_ is the Vogel temperature. (Fig. 4B)

As shown in Table 1, NPTAm-3 showed the highest ionic conductivity (*σ*_DC_ at 30 °C) of 5.1 × 10^−7^ S cm^−1^ compared with NPTAm-1 (2.8 × 10^−8^ S cm^−1^) and NPTAm-2 membranes (7.9 × 10^−8^ S cm^−1^). The enhanced ionic conductivity of NPTAm-3 could be attributed to the high dissociation and mobility of the maximum TFSI^−^ anions promoted by the largest number of side-chain OEG as discussed above on *T*_g_s. Except from the crosslinked polyether-based TPIL (*σ*_DC_ up to 3.9 × 10^−6^ S cm^−1^),^9^ the ionic conductivity of NPTAm-3 is slightly high for a reported crosslinked TPILs (*σ*_DC_ ranges from 2.2 × 10^−11^ to 2.0 × 10^−7^ S cm^−1^),^14,16,23^ clearly demonstrating the structural advantage of introducing the conductive ions in the side groups spaced by flexible ether groups with TFSI^−^ as counter-anions.

### Gas separation of the NPTAm membranes

**Fig. 5:**
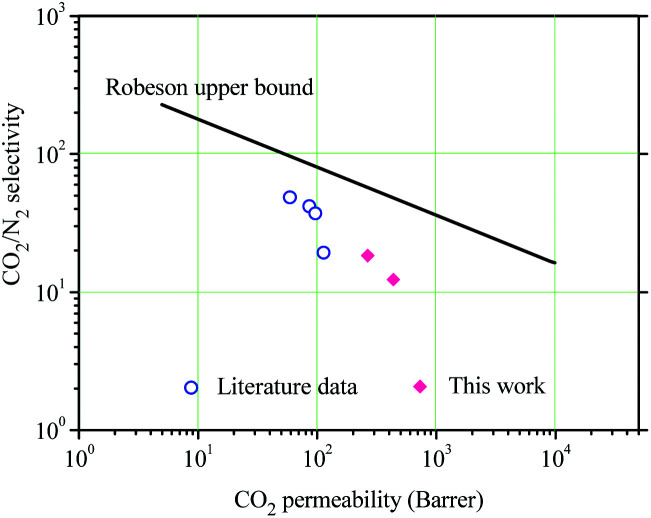
Comparison of the gas transport properties of the membranes with the literature data.^9,24^

The gas separation performances of the NPTAm membranes are summarized in Table 2. The membranes show CO_2_ permeability of 264.7–434.5 barrer and CO_2_/N_2_ selectivity of 18.4–12.3. Compared to NPTAm-1, NPTAm-3 had higher CO_2_ permeability but decreased CO_2_/N_2_ selectivity, following a traditional trade-off. Fig. 5 shows the comparison of the separation performances of this work with the reported data of other crosslinked TPILs membranes.^9^ The CO_2_ permeability is relatively enhanced. Firstly, there were higher density of side-chain OEG and 1,2,3-triazolium moieties inside the NPTAm membranes. The numerous OEG provided plentiful polar ether groups, which are efficient CO_2_-philic units and could efficiently improve CO_2_ affinity. Secondly, the presence of aromatic groups in the backbone significantly improves the CO_2_ uptake.^6^ Although both work could not reach Robeson's upper bound, they convince crosslinked TPILs membranes as promising separation materials and optimization of their structure through Click chemistry (1,3-dipolar cycloaddition reaction) will endow them further enhancement in both the permeability and selectivity.

**Table 2:** Gas transport properties of the NPTAm membranes^a^

Entry	*P*_CO_2__ (barrer)	*P*_N_2__ (barrer)	CO_2_/N_2_ selectivity
NPTAm-1	264.7	14.4	18.4
NPTAm-3	434.5	35.1	12.3

a 1 barrer = 10^−10^ cm^3^ (STP) cm (cm^2^ s cmHg)^−1^.

## Conclusion

Crosslinked poly(1,2,3-triazolium)s membranes with 1,2,3-triazolium in side chain spaced by oligo(ethylene glycol) were prepared through “Click” chemistry (Huisgen 1,3-dipolar cycloaddition reaction). These self-standing membranes show good thermal properties with *T*_g_s ranging from −1.0 to −7.5 °C and *T*_d10_ above 330 °C. These membranes have good ionic conductivity with *σ*_DC_ at 30 °C up to 5.1 × 10^−7^ S cm^−1^. The structure of rigid benzene ring in the backbone with 1,2,3-triazolium spaced by flexible OEG as side groups contribute to enhanced CO_2_ permeation, up to 434.5 barrer at 4 atm pressure.

## Conflicts of interest

There are no conflicts to declare.

## Supplementary Material

RA-008-C8RA00541A-s001
