# Supplementary material for: Novolac-based poly(1,2,3-triazolium)s with good ionic conductivity and enhanced CO2 permeation
Source: RSC Adv. 2018 Feb 26;8(16):8552–7. doi: 10.1039/c8ra00541a (PMC9078539; doi:10.1039/c8ra00541a)
Supplement: RA-008-C8RA00541A-s001 [file RA-008-C8RA00541A-s001.pdf]

Electronic Supplementary Material (ESI) for RSC Advances.
This journal is © The Royal Society of Chemistry 2018

Supporting information for: Novolac-based poly(1,2,3-triazolium)s with good ionic conductivity and enhanced CO_2 permeation

Lvyuan Ye, Liqiang Wan, Junkun Tang, Yujing Li, Farong Huang*

Key Laboratory of Specially Functional Polymeric Materials and Related Technology, School of Materials Science and Engineering, East China University of Science and Technology, Ministry of Education, Shanghai 200237, China.

Preparation of the crosslinked NPTAm-2 membrane

Synthesis of PN-OEG through CuAAC. To a solution of PN resin (3.17 g, 20 mmol of alkyne group) and OEG-N_3 (2.09 g, 12 mmol) in DMF (50 mL), $CuSO_4 \cdot 5H_2O$ aqueous solution (0.8 mmol in 1.2 mL of H_2O) was added. After bubbling nitrogen for 30 min, fresh sodium ascorbate solution (2.4 mmol in 0.8 mL H_2O) was added. The resulting mixture was stirred for 48 h at 55 °C. After evaporation of the solvent under reduced pressure, the mixture was poured into H_2O, then extracted with DCM (50 mL), and the organic layer was washed with EDTA aqueous solution and saturated NaCl aqueous solution until the aqueous phase became colorless. After dried by $MgSO_4$, the organic layer was concentrated and precipitated three times with ether, then dried in vacuum and a reddish brown solid **PN-OEG** (4.52 g, yield 86%) was obtained.

Synthesis of $[PN\text{-}OEG]^+I^-$. To a solution of **PN-OEG** (1.31 g, 3 mmol of triazole groups) in 50 mL CH_3CN, CH_3I (1.42 g, 10 mmol) was added, and the mixture was stirred at 45 °C for 3 d. The mixture was concentrated and precipitated three times in diethyl ether and dried in vacuum to get **$[PN\text{-}OEG]^+I^-$** (1.67 g, yield 96%) as a yellow

solid.

Synthesis of $[PN\text{-}OEG]^+TFSI^-$. A solution of **$[PN\text{-}OEG]^+I^-$** (1.45 g, 2.5 mmol of 1,2,3-triazole groups) and $LiNTf_2$ (2.15 g, 7.5 mmol) in a mixture of acetone (35 mL) and methanol (35 mL) was stirred at 45 °C for 2 d. The heterogeneous mixture was concentrated and precipitated in deionized water several times, until there was no AgI precipitate when the deionized water was tested with $AgNO_3$, then dried in vacuum, and a reddish brown viscous material **$[PN\text{-}OEG]^+TFSI^-$** (1.10 g, yield 60%) was obtained.

Preparation of the crosslinked NPTAm-2 membrane. A stoichiometric mixture of **$[PN\text{-}OEG]^+TFSI^-$** (2.2 g, 2 mmol of alkyne) and *p*-xylyene diazide (0.19 g, 1 mmol) was dissolved in DMF (6 g), and then was stirred at 70 °C for 2 h, following by casting onto a glass plate with a stainless steel scraper. The glass plate with casting dispersion was placed onto a horizontal platform in a heating oven, which had been preheated to 70 °C. Then, the system was sequentially cured during 3 h at 70 °C, 6 h at 80 °C, 2 h at 120 °C and 4 h at 150 °C. After that, the heating oven was turned off and the whole system was gradually cooled to room temperature. The membrane, named as **NPTAm-2**, was removed by immersing the glass plate in water and was then dried at 100 °C for 0.5 h for further use.

Preparation of the crosslinked NPTAm-3 membrane

Synthesis of PN-OEG through CuAAC. To a solution of PN resin (3.17 g, 20 mmol of alkyne group) and $OEG\text{-}N_3$ (2.78 g, 16 mmol) in DMF (50 mL), $CuSO_4 \cdot 5H_2O$ aqueous solution (0.8 mmol in 1.2 mL of H_2O) was added. After bubbling nitrogen for 30 min, fresh sodium ascorbate solution (2.4 mmol in 0.8 mL H_2O) was added. The resulting mixture was stirred for 48 h at 55 °C. A reddish brown solid **PN-OEG** (4.94 g, yield 83%) was obtained through the same after-treatment procedure.

Synthesis of $[PN\text{-}OEG]^+I^-$. To a solution of **PN-OEG** (1.12 g, 3 mmol of triazole groups) in 50 mL CH_3CN, CH_3I (1.42 g, 10 mmol) was added, and the mixture was stirred at 45 °C for 3 d. The mixture was concentrated and precipitated three times in diethyl ether and dried in vacuum to get **$[PN\text{-}OEG]^+I^-$** (1.42 g, yield 92%) as a yellow solid.

Synthesis of $[PN\text{-}OEG]^+TFSI^-$. A solution of **$[PN\text{-}OEG]^+I^-$** (1.29 g, 2.5 mmol of 1,2,3-triazole groups) and $LiNTf_2$ (2.15 g, 7.5 mmol) in a mixture of acetone (35 mL) and methanol (35 mL) was stirred at 45 °C for 2 d. The heterogeneous mixture was concentrated and precipitated in deionized water several times, until there was no AgI precipitate when the deionized water was tested with $AgNO_3$, then dried in vacuum, and a reddish brown viscous material **$[PN\text{-}OEG]^+TFSI^-$** (1.03 g, yield 62%) was obtained.

Preparation of the crosslinked NPTAm-3 membrane. A stoichiometric mixture of **$[PN\text{-}OEG]^+TFSI^-$** (5.34 g, 2 mmol of alkyne) and *p*-xylyene diazide (0.19 g, 1 mmol) was dissolved in DMF (13 g), and then was stirred at 70 °C for 2 h, following by casting onto a glass plate with a stainless steel scraper. The glass plate with casting dispersion was placed onto a horizontal platform in a heating oven, which had been preheated to 70 °C. Then, the system was sequentially cured during 3 h at 70 °C, 6 h at 80 °C, 2 h at 120 °C and 4 h at 150 °C. After that, the heating oven was turned off and the whole system was gradually cooled to room temperature. The membrane, named as **NPTAm-3**, was removed by immersing the glass plate in water and was then dried at 100 °C for 0.5 h for further use.

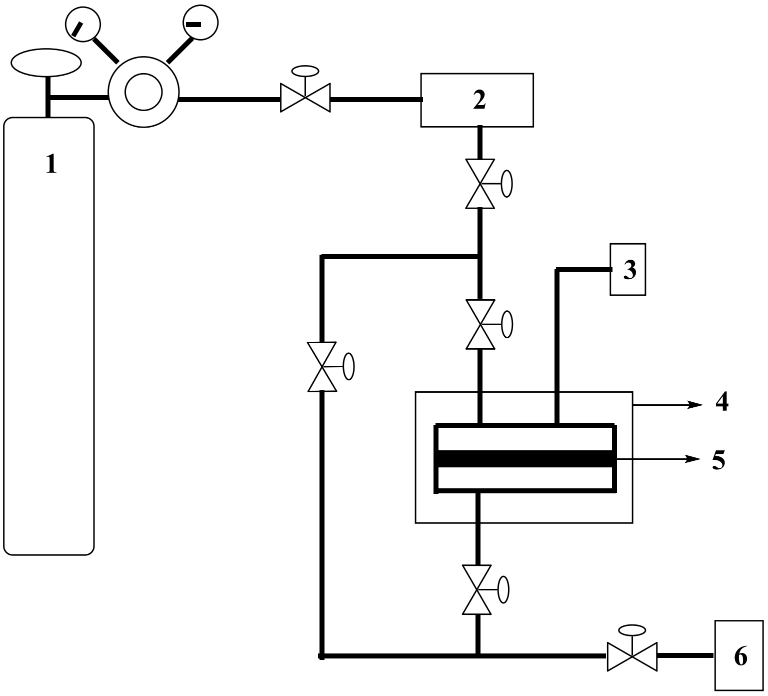

1: Gas supplier
2: Mass flow controller
3: Gas flowmeter
4: Constant temperature chamber
5: Permeation cell
6: Gas flowmeter

Fig. S1 A schematic of the permeation set-up

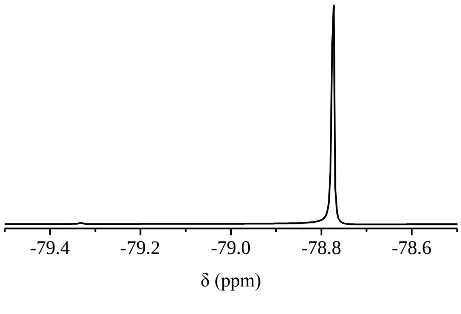

Fig. S2 ^{19}F NMR spectra of $[PN\text{-}OEG]^{+}TFSI^{-}$.

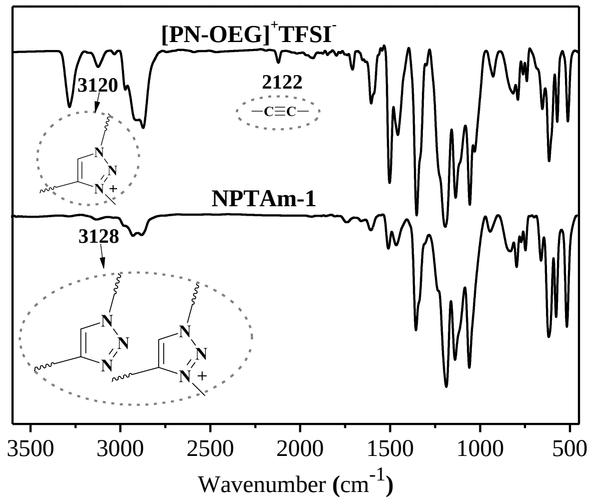

Fig. S3 FTIR spectra of $[PN\text{-}OEG]^{+}TFSI^{-}$ and NPTAm-1.

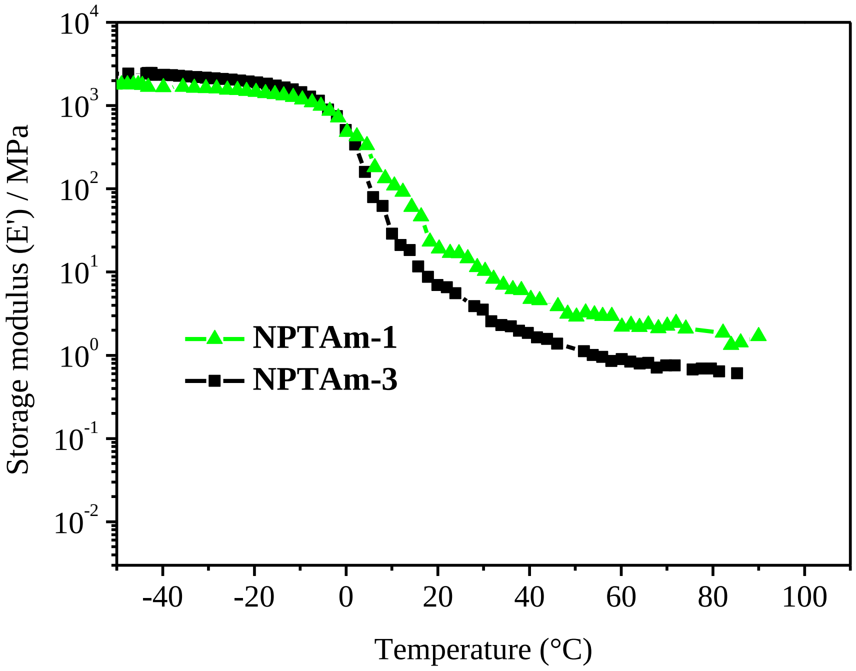

Fig. S4 DMA curves of the crosslinked NPTAm membranes.

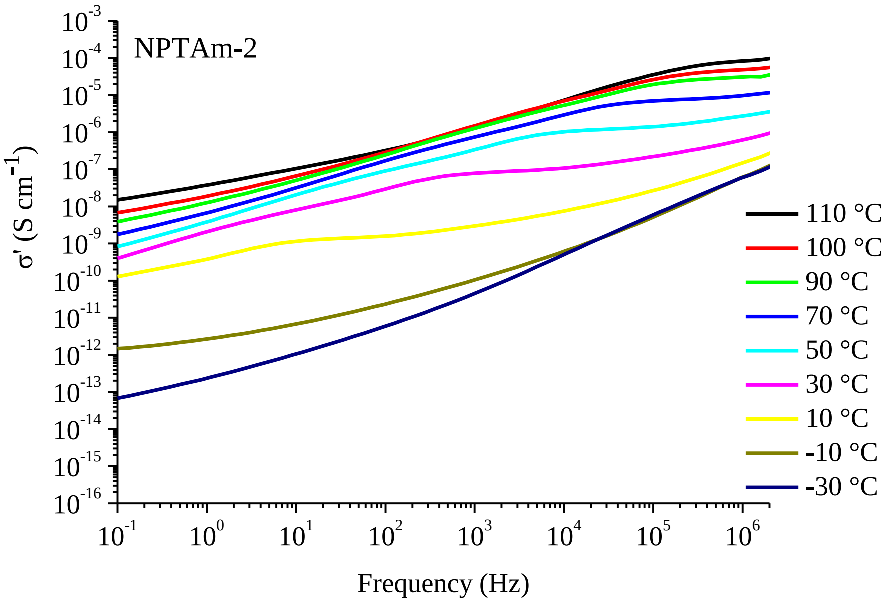

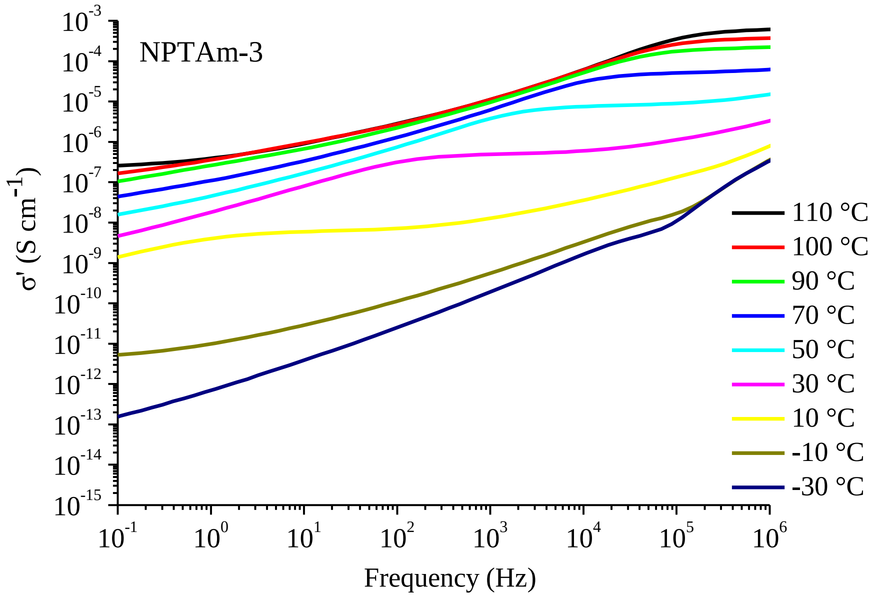

Fig. S5 Conductivity versus frequency for the crosslinked NPTAm membranes.
